# Bibliographical Department

**Published:** 1881-12

**Authors:** 


### BIBLIOGRAPHICAL DEPARTMENT.

WALSH'S PHYSICIANS' CALL-BOOK AND TABLET. WALSH'S PHYSICIANS' HANDY LEDGER.—These two ingeniously arranged works have been devised by the publisher, Dr. Ralph Walsh, of Washington, to decrease the labor and drudgery of keeping the accounts of a large practice. That he has successfully accomplished this laudable intention is evidenced by the popularity which the *Call-Book* and *Ledger* have attained among the profession.

THE CENTURY MAGAZINE (*Scribner's Monthly*), Vol. XXIII., Nos. 1 & 2, November and December, 1881.

We take great pleasure in adding this sterling magazine to our exchange list. Under its new name it promises to become even a more welcome visitor than it was under its old name of *Scribner's Monthly*. The first two numbers of the new volume contain portraits of George Eliot, Salvini, the great tragedian, as Macbeth, Dr. J. G. Holland, the late editor of the magazine, and of the late President Garfield. The poems, stories, literary and descriptive articles contained in it are of the highest class, and the wood-cuts are of a quality not surpassed by any similar publication in the world. The subscription price of the *Century* is $4.00 a year. We will send it and the INDEPENDENT PRACTITIONER to one address for $6.00 a year.

*Atresia of the Vagina and Uterus.* By A. F. Erich, M. D., professor of diseases of women, College of Physicians and Surgeons, Baltimore. Reprint from *Atlanta Medical Register*, November, 1881.

*How to use the Bromides.* By George M. Beard, A. M., M. D. Reprint from the *Journal of Nervous and Mental Disease*, July, 1881. An excellent practical paper, written in Dr. Beard's entertaining manner.

*The Treatment of Hydrocele and Serous Cysts in General, by the injection of Carbolic Acid.* By R. J. Levis, M. D. Reprint from Trans. Med. Society of Penna., 1881.

*Deuxieme Circulaire Annuelle de l' Hopital Dentaires Libres de Paris. Annee Scolaire 1881-1882.* The faculty of instruction of this school consists of eight professors. The chair of operative dentistry, we notice, is filled by Dr. Levett, "*de New York.*"

*Reglement de l' Ecole Dentaire de Geneve* has also been received. The school has just been organized, and its first course of lectures and demonstrations commenced on October 21st. The professor of mechanical dentistry is Dr. J. Marcelin, also of the *College de New York.* Evidently the practical knowledge of dentistry imparted by our American colleges is appreciated at its just value by our trans-Atlantic brethren.

*Correspondenz-Blatt fuer Zahnærzte*, Bd. X, Heft IV, Berlin, October, 1881, contains several interesting original communications, an abstract of dental society work, including the International Congress, and the Ohio Dental Society, and a number of interesting selections from current dental literature.

*The Annals of Anatomy and Surgery* has just been received, and placed on our exchange list. It is a journal whose handsome exterior is equalled by the solidity of its contents.

*New Exchanges.*—Since our last issue we have received and placed upon our list of regular exchanges the *Therapeutic Gazette*, the *New England Medical Monthly*, the *St. Louis Courier of Medicine*, the *Chicago Medical Journal and Examiner*, the *Medical News and Abstract*, and that solidest of all American quarterlies, the *American Journal of the Medical Sciences.*

